# Necroptosis induced by ruthenium (II) complexes as mitochondrial disruptors

**DOI:** 10.1038/s41420-024-02033-z

**Published:** 2024-05-28

**Authors:** Joana Gonçalves, Joana D. Amaral, Rita Capela, Maria de Jesus Perry, Cláudia Braga, Maria Manuela Gaspar, Fátima M. Piedade, Lubertus Bijlsma, Antoni Roig, Sandra N. Pinto, Rui Moreira, Pedro Florindo, Cecília M. P. Rodrigues

**Affiliations:** 1 Research Institute for Medicines (iMed.ULisboa), Faculty of Pharmacy, Universidade de Lisboa, Lisbon, Portugal; 2 Instituto de Biofísica e Engenharia Biomédica, Faculty of Sciences, Universidade de Lisboa, Lisbon, Portugal; 3 Departamento de Química e Bioquímica, Faculty of Sciences, Universidade de Lisboa, Lisbon, Portugal; 4 Centro de Química Estrutural, Institute of Molecular Sciences, Instituto Superior Técnico, Universidade de Lisboa, Lisbon, Portugal; 5 Environmental and Public Health Analytical Chemistry, Research Institute for Pesticides and Water, University Jaume I, Castelló, Spain; 6 iBB-Institute for Bioengineering and Biosciences, Department of Bioengineering, Instituto Superior Técnico, Universidade de Lisboa, Lisbon, Portugal

**Keywords:** Drug development, Screening

## Abstract

Inducing necroptosis in cancer cells has emerged as an effective strategy to overcome drug resistance. However, while organic small molecules have been extensively studied for this purpose, metal-based compounds have received relatively little attention as triggers of necroptosis. The development of ruthenium (II) hybrid compounds, particularly those containing triazene (Ru-TRZ), highlights a novel avenue for modulating necroptotic cell death. Here we show that incorporating a methyltriazene moiety, a known alkylating warhead, confers superior mitochondrial-targeting properties and enhances cell death compared to amide-containing counterparts. Ru-hybrid TRZ2 exhibits also antitumor efficacy against in vivo drug-resistant cancer cells. Mechanistically, we demonstrate that Ru-TRZ hybrids induce apoptosis. In addition, by activating downstream RIPK3-driven cell death, TRZ2 proficiently restrains normal mitochondrial function and activity, leading to cancer cell necroptosis. Finally, TRZ2 synergizes anti-proliferative activity and cell death effects induced by conventional drugs. In conclusion, Ru-TRZ2 stands as a promising ruthenium-based chemotherapeutic agent inducing necroptosis in drug resistant cancer cells.

## Introduction

Cancer ranks as a leading cause of death worldwide and a major barrier to increased life expectancy [1]. While surgery is the only curative option, chemotherapy and radiotherapy are still the golden standard therapeutic approaches in cancer. One of the major challenges in cancer treatment is cancer cell drug resistance, which often leads to therapeutic failure [2]. Effective therapeutic strategies should target resistance mechanisms such as evasion of cell death, which allow cancer cells to tolerate adverse or stressful conditions [3].

Metal-based drugs have been used as chemotherapeutics. Platinum-based anticancer agents, such as cisplatin, despite high therapeutic effectiveness in various types of tumors, induce undesirable side effects in patients. To overcome these complications, a second generation of platinum-based drugs including oxaliplatin, was developed [4]. However, over time many successfully administered platinum drugs lost potency, due to acquired resistance, leading to seek and develop new efficacious and safe metal-based therapeutics, resorting to other transition metals such as ruthenium (Ru). Ru compounds, due to their ability to mimic iron, proved to be promising chemotherapeutic agents due to reduced toxicity and good selectivity for tumors [5]. Ru complexes have multiple molecular targets and various mechanisms of anti-tumor activity [5], with the ability to interact directly with DNA and impact on DNA replication and transcription, much like the commonly used platinum-based drugs. These compounds also show good topoisomerase inhibitory activity [6, 7] and can accumulate in mitochondria, endoplasmic reticulum, lysosomes, and the cell nucleus [8, 9]. In fact, the mitochondria seem to be the primary therapeutic target of Ru complexes, considering that several studies report mitochondrial dysfunction and associated cell death by apoptosis [10–12].

Necroptosis is a form of regulated cell death that serves as an alternative mode of regulated cell death overcoming apoptosis resistance. Necroptosis is caspase-independent, triggered by the activation of death receptors, such as the tumor necrosis factor receptor 1. When caspase-8 is inhibited, the receptor-interacting protein kinase 1 (RIPK1) is recruited to form the necrosome with RIPK3. Downstream of RIPK1 phosphorylation, RIPK3 is activated by autophosphorylation and then phosphorylates mixed lineage kinase domain-like pseudokinase (MLKL), which mediates plasma membrane permeabilization, leading to cell lysis as a result of the osmotic imbalance [13, 14].

Reports on metal-based chemotherapeutic agents with the ability to induce necroptosis are rare. Cisplatin can trigger necrosis in cancer cells, in the presence of a caspase inhibitor, indicating that necroptosis is optimally induced when the apoptotic machinery is compromised [15, 16]. Recently, two Rhenium(V)-oxo complexes were developed as necroptosis triggers and demonstrated excellent therapeutic efficiency [17]. Furthermore, Zn(II), Ni(II), Os(II), and Fe(III) complexes were also utilized to induce necroptosis in cancer cells [18, 19]. Recently, a ruthenium (II) complex, dual inhibitor of topoisomerase I and II, was identified as potential chemotherapeutic agent with ability to induce necroptosis [7]. The major novelty was the PARP-1 activation of the necroptotic machinery. Here, we synthesize a new family of ruthenium (II)-triazene hybrids (Ru-TRZ) and dissected the overall cell death mechanisms, focusing on the ability to develop potent necroptosis activators as mitochondrial disruptors in cancer cells with apoptosis-resistant phenotypes.

## Results

### Synthesis and biological activity evaluation of Ru-TRZ hybrids

**Fig. 1:**
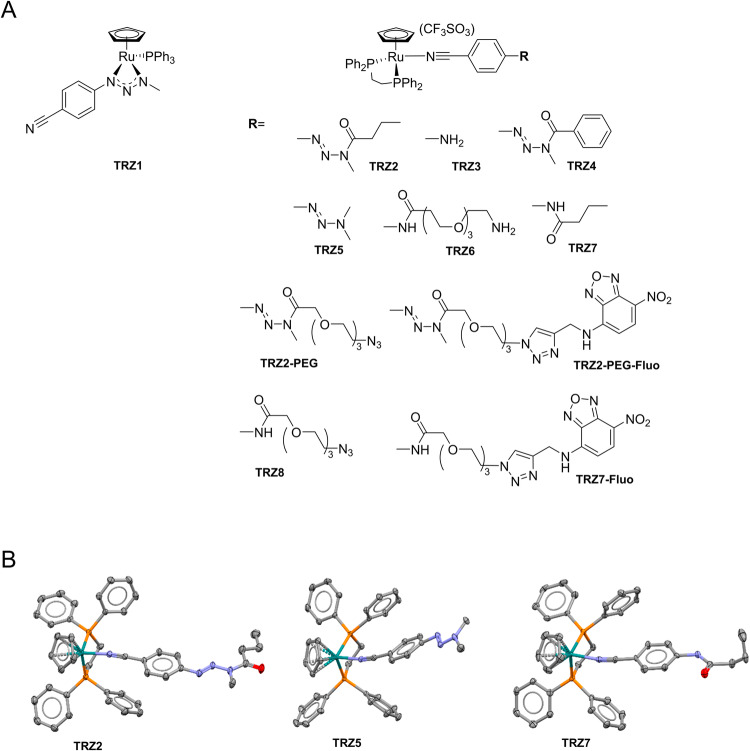
Chemical structure of Ru-TRZ hybrids. **A** Organoruthenium(II) compounds TRZ1-11. **B** X-ray structure of monocationic complexes TRZ2, TRZ5, and TRZ7. Hydrogen atoms were omitted for clarity.

Ru-TRZ1 was synthesized by generation of 1-(4-cyanophenyl)-3-methyltriazenium (L1) with sodium hydride, and reaction with [(η^5^-C_5_H_5_)Ru(PPh_3_)_2_]^+^ trifluoromethanesulphonate salt generated in situ from the parent chlorinated compound. Complexes Ru-TRZ (Fig. 1A) were synthesized by chloride abstraction from [(η^5^-C_5_H_5_)RuCl(Dppe)] with silver trifluoromethanesulphonate in presence of the corresponding nitrile ligand. Organometallic compounds were characterized by ^1^H-NMR and HRMS; compounds TRZ2, TRZ5, and TRZ7 were further characterized by single crystal SCXRD, confirming the usual three-legged piano stool geometry around the Ru center (Fig. 1B) [20].

The antiproliferative potential of Ru-TRZ was first evaluated in HCT116 and HT29 human colon cancer cells. All compounds strongly inhibited cell proliferation with IC_50_ values ranging between 0.01 and 4 µM (Table 1), with the exception of TRZ1. The modified forms of TRZ2, TRZ2-PEG with a polyethylene glycol (PEG) chain to improve solubility, and TRZ2-PEG-Fluo, a TRZ2 pegylated analog incorporating an NBD-fluorescent tag, were similarly strong inhibitors of cell proliferation. Of note, the elimination of Ru atom in the chemical structure of TRZ7-Fluo and TRZ2-PEG-Fluo complexes resulted in lack of activity (IC50 > 200 µM) (data not shown). Similarly, in CCD-18Co human colon fibroblasts, all tested compounds displayed IC_50_ values > 100 µM, indicating selectivity against cancer cells (data not shown).

**Table 1:** IC_50_ of Ru-TRZ hybrids in HCT116 and HT29 cells.

Ru-TRZ	IC_50_ (μM) HCT116	95% CI ^a^	IC_50_ (μM) HT29	95% CI^a^
TRZ1	16.75	11.12–25.21	34.78	21.49–56.30
TRZ2	0.45	0.31–0.66	0.24	0.21–0.28
TRZ3	0.46	0.36–0.57	0.20	0.15–0.27
TRZ4	0.55	0.44–0.69	0.23	0.18–0.29
TRZ5	0.22	0.15–0.32	0.31	0.17–0.59
TRZ6	2.42	2.12–2.77	3.54	2.51–4.98
TRZ7	0.55	0.34–0.89	0.36	0.27–0.53
TRZ8	0.46	0.31–0.70	0.27	0.18–0.40
TRZ2-PEG	0.29	0.27–0.32	0.38	0.29–0.49
TRZ2-PEG-Fluo	0.30	0.28–0.33	0.48	0.38–0.62
TRZ7-Fluo	1.83	1.39–2.40	2.19	1.59–3.04

IC_50_ determined by the MTS assay after 72 h of compound incubation. Each value is the mean of three independent experiments performed in duplicate.

^a^95% confidence interval.

Based on potency and drug-like properties, TRZ2 and TRZ7 (amide counterpart) were selected for further studies. We also selected TRZ2-PEG and TRZ2-PEG-Fluo.

The cytotoxic effects of these compounds were further evaluated in HT29 human colorectal adenocarcinoma cells, HepG2 human hepatoma cells and AML12 mouse hepatocytes, by measuring AK release after 24 h of compound incubation (Supplementary Fig. 15). As depicted in Table 2, TRZ2-PEG was the most active compound with IC_50_ values of 2.19 and 5.85 µM in HepG2 and HT29 cells, respectively. Importantly, TRZ2-PEG cytotoxic activity is considerably lower in AML12 hepatocytes with 27.05 µM IC_50_, suggesting a selective effect of the compound towards cancer cells. TRZ2-PEG-Fluo and TRZ7 presented similar effects in both colon and hepatic cancer cell lines, while TRZ2 was only active in HT29 cells.

**Table 2:** Cytotoxic activity of TRZ2, TRZ7, TRZ2-PEG and TRZ2-PEG-Fluo in HT29, HEPG2 and AML12 cells.

Ru-TRZ	HT29 EC_50_ (μM)	95% Cl^a^	HEPG2 EC_50_ (μM)	95% Cl^a^	AML12 EC_50_ (μM)	95% Cl^a^
TRZ2	7.66	6.45–9.10	35.30	31.67–39.79	18.19	12.89–25.47
TRZ7	15.20	12.15–19.09	16.75	14.52–19.32	40.22	24.30–115.4
TRZ2-PEG	5.85	3.88–8.69	2.19	0.91–4.14	27.05	2.512–61.57
TRZ2-PEG-Fluo	18.85	15.04–23.24	16.23	13.70–19.16	35.59	28.06–50.27

IC_50_ determined by AK release after 24 h compound incubation. Each value is the mean of three independent experiments performed in duplicate.

^a^95% confidence interval.

### Ru-TRZ hybrids enter cancer cells and accumulate in mitochondria

**Fig. 2:**
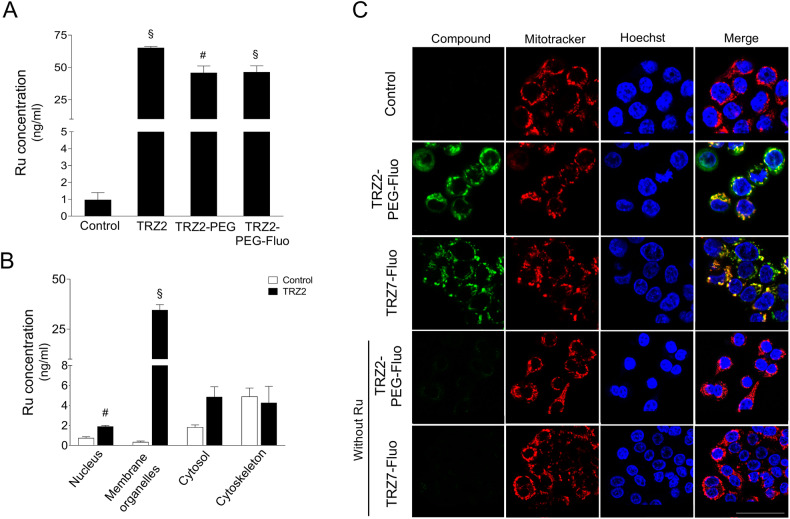
Ru-TRZ hybrids enter HT29 cancer cells and accumulate preferentially in mitochondria. Ruthenium (Ru) concentration was determined by ICP-MS in (**A**) whole-cell lysates and (**B**) subcellular fractions nucleus, membrane organelles, cytosol, and cytoskeleton isolated from HT29 cells exposed to 5 µM of indicated Ru-TRZ compounds or DMSO (vehicle control), for 24 h. **C** Representative confocal images of HT29 cells exposed to 1 µM of indicated fluorescently-tagged Ru-TRZ hybrids (green) or DMSO (vehicle), for 15 min, and stained with mitotracker (red). Nuclei were counterstained with Hoechst 33342 (blue). Scale bar, 25 µm. Results are expressed as Ru concentration (ng/ml) for the mean ± SEM of three independent experiments. Statistical analysis was performed using Student’s *t*-test and comparing Ru-TRZ-exposed cells with controls. ^#^*p* < 0.05 and ^§^*p* < 0.001 from control cells.

To elucidate if Ru-TRZ were able to enter cancer cells and if the amount of compound uptaken was correlated with their cytotoxic properties, quantitative determination of Ru inside the cells was performed by inductively coupled plasma-mass spectrometry (ICP-MS). The total levels of Ru detected in whole-cell extracts from HT29 cells exposed to TRZ2, TRZ2-PEG and TRZ2-PEG-Fluo increased more than 50-fold relative to vehicle control (Fig. 2A). The three complexes were equally uptaken, indicating that conjugation with PEG and fluorescent groups did not interfere with TRZ2 cellular uptake. In addition, analysis of the subcellular fractions suggests that TRZ2 accumulated preferentially in membrane organelles (Fig. 2B). Considering these results and previous publications showing that Ru complexes are usually targeted to mitochondria [21, 22], we examined the subcellular localization of TRZ2-PEG-Fluo and TRZ7-Fluo that correspond to the fluorescently-tagged versions of TRZ2-PEG and TRZ7, respectively. Confocal microscopy imaging of HT29 cells clearly revealed that after a 15 min-period of incubation, both TRZ2-PEG-Fluo and TRZ7-Fluo colocalized with mitochondria with a Pearson’s correlation coefficient of 0.839 and 0.869, respectively (Fig. 2C). Importantly, organic ligands TRZ2-PEG-Fluo and TRZ7-Fluo without Ru atom were not detected intracellularly, showing that the metal is the driving force for accumulation of these compounds inside the cells and within mitochondria.

### Ru-TRZ hybrids induce mitochondrial depolarization and apoptotic cell death

**Fig. 3:**
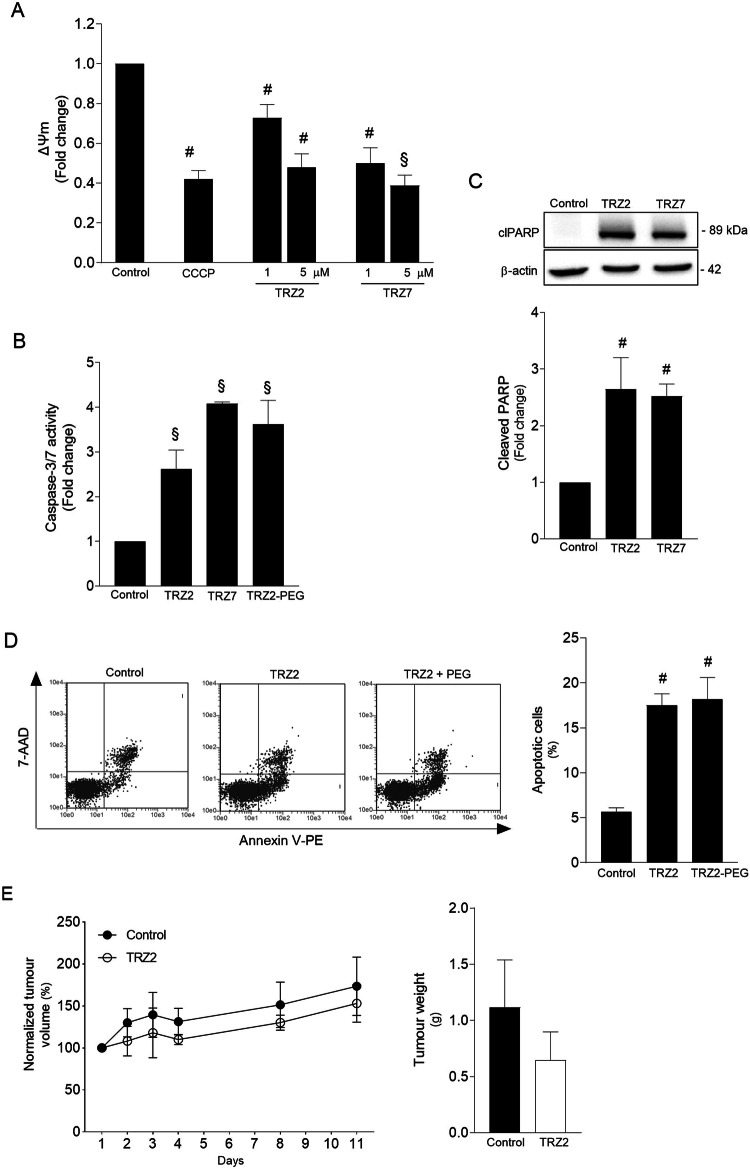
Ru-TRZ hybrids induce mitochondrial depolarization and apoptotic cell death. Ru-TRZ effects were determined in HT29 cells and HT29 xenograft tumor-bearing mice. **A** Mitochondria transmembrane potential (ΔΨ_m_) was evaluated by JC-1 staining of HT29 cells exposed to TRZ2 and TRZ7 for 2 h or CCCP (200 µM) for 4 h (positive control). Data presented correspond to the ratio of fluorescence emitted at 590 and 530 nm. **B** Caspase-3/7 activity was determined in HT29 cells exposed to 10 μM of indicated Ru-TRZ compounds for 24 h, using the Caspase-Glo 3/7 assay. **C** Representative immunoblot of cleaved PARP-1 (clPARP) in whole-cell extracts from HT29 cells exposed to indicated Ru-TRZ compounds for 24 h and respective quantification normalized to endogenous β-actin. **D** Apoptotic cell death was analyzed by flow cytometry using the Guava Nexin assay, following incubation of HT29 cells with indicated Ru-TRZ compounds (10 µM), or vehicle, for 24 h. Representative flow cytometry plots (*left*) and respective quantification (*right*). Results are expressed as mean ± SEM from at least three independent experiments. **E** Normalized tumor volume evolution of TRZ2- and vehicle-treated HT29 xenograft tumor-bearing mice. Line graph represents tumor volume normalized to baseline at day 1 (*left*). Bar graph represents tumor weight after 20 days of treatment with TRZ2 or vehicle control (*right*). Results are expressed as mean ± SEM of the tumor volume or weight of at least three animals in each group. DMSO is the vehicle control. ^#^*p* < 0.05 and ^§^*p* < 0.001 from control cells; *p < 0.05 and ^$^*p* < 0.001 from Ru-TRZ-treated cells.

Given that our Ru-TRZ complexes preferentially targeted mitochondria, we used the JC-1 dye to assess ΔΨm, which represents an essential parameter of healthy mitochondrial function. Exposure of cells to 1 µM TRZ2 and TRZ7 for 2 h, induced a significant loss of ΔΨm (*p* < 0.05). When compound concentration increased to 5 µM, we observed a dose-dependent effect with a loss of ΔΨm similar to the one induced by CCCP, a well-known depolarizing agent (Fig. 3A). We also detected an increase of up to 4-fold in caspase-3/7 activity in cells treated with TRZ2, TRZ2-PEG and TRZ7 (Fig. 3B; *p* < 0.001), and subsequent cleavage of PARP-1 (Fig. 3C; *p* < *0.05*). Both mitochondrial dysfunction and caspase activation are important hallmarks of apoptotic cell death. The double staining method with Annexin V-FITC and 7-Aminoactinomycin D (7-AAD) followed by flow cytometry analysis confirmed these findings. As illustrated in Fig. 3D, treatment of HT29 cells with Ru-TRZ resulted in induction of apoptosis as revealed by a 10–15% increase of cells undergoing early phases of apoptosis. Overall, our data suggest that Ru-TRZ cytotoxic effects are primarily mediated by the activation of the intrinsic pathway of apoptosis.

### Antitumour activity of Ru-TRZ hybrids in HT29 xenograft mice

To confirm TRZ2 antitumour activity, we performed a pilot study using cell death resistant HT29 xenograft tumor-bearing mice, before proceeding to future hit optimization studies. The HT29 cell line was selected for its known cell death resistance phenotype. Mice from test group were treated intravenously every 2 days (in a total of 10 injections) with 5 mg/kg of TRZ2 while control animals received DMSO. The results indicated that tumor burden tended to decrease in TRZ2-treated animals compared to the control group. Over time, tumor volumes were consistently smaller, and the weights at the time of sacrifice were also reduced (Fig. 3E), thus confirming the in vivo efficacy of TRZ2.

### Ru-TRZ hybrids are potent inducers of necroptosis

**Fig. 4:**
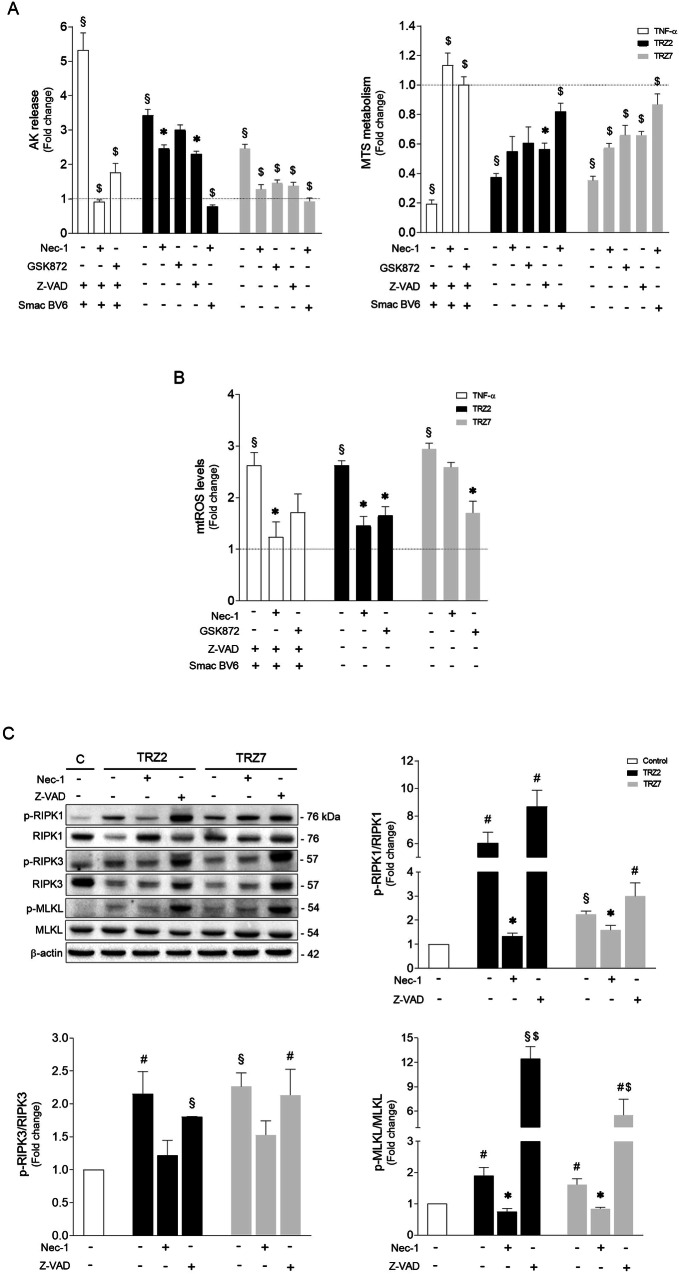
Ru-TRZ hybrids induce necroptotic cell death that is exacerbated when apoptosis is compromised. **A** HT29 cells were treated with indicated Ru-TRZ (10 µM), with or without Nec-1 (necroptosis inhibitor), GSK872 (RIPK3 inhibitor), and Z-VAD (pan-caspase inhibitor). Human TNF-α (20 ng/ml) plus Z-VAD and Smac mimetic is the positive control for necroptosis induction. Cell death and cell viability were assessed 24 h after compound incubation using a luminescence-based readout for AK release (*left*), and a colorimetric-based readout for MTS metabolism (*right*). **B** Mitochondrial ROS was determined in HT29 cells exposed to indicated Ru-TRZ compounds for 15 min by fluorometric measurement of MitoSOX Red (580 nm). **C** Representative immunoblots of necroptosis mediators RIPK1, RIPK3 and MLKL, and respective phosphorylated forms, p-RIPK1, p-RIPK3 and p-MLKL, in whole-cell extracts from HT29 cells exposed to indicated Ru-TRZ compounds for 24 h (*top left*), and quantification of p-RIPK1/RIPK1 (*top right*), p-RIPK3/RIPK3 and p-MLKL/MLKL ratios (*bottom*). Blots were normalized to endogenous β-actin. Results are expressed as mean ± SEM fold-change to control, from at least three independent experiments. DMSO is the vehicle control. ^#^*p* < 0.05 and ^§^*p* < 0.001 from control vehicle-treated cells; *p < 0.05 and ^$^*p* < 0 .001 from cells exposed to Ru-TRZ alone.

One of the hallmarks of human cancers is resistance to apoptotic cell death [23]. Moreover, evasion of apoptosis may contribute to carcinogenesis, tumor progression, and treatment resistance [24]. Therefore, therapeutic activation of other forms of cell death, such as regulated necrosis, or necroptosis, may be an alternative or complementary approach to treat cancer [16]. Consequently, we hypothesized that Ru-TRZ-mediated cytotoxicity involves necroptosis, particularly in cases where apoptosis is compromised. As expected, exposure of HT29 cells to TNF-α plus the pan-caspase inhibitor Z-VAD and Smac mimetic BV6 (TSZ), a well-described stimulus of necroptosis, induced severe cell damage that was rescued by RIPK1 chemical inhibitor, Nec-1 (Fig. 4A). Inhibition of RIPK3 by GSK872 also significantly decreased TSZ-induced AK release (Fig. 4A). Similarly, exposure of cells to 10 µM TRZ2 and TRZ7 led to high levels of cell death that was partially reverted by Z-VAD, but also by Nec-1 and GSK782, suggesting that both apoptosis and necroptosis are activated (Fig. 4A). These results were corroborated by the MTS metabolism assay, in which Ru-TRZ treatment resulted in a significant decrease of cell viability that, in turn, was partially rescued by both Z-VAD and Nec-1 (Fig. 4A). Concomitant incubation of both inhibitors reverted Ru-TRZ-cytotoxic effects nearly to control levels (Fig. 4A; *p* < *0.001*), which further supports the dual effect of Ru-TRZ compounds. L929 cells were equally sensitive to Ru-TRZ treatment (Supplementary Fig. 16). Mitochondrial stress accompanied by ROS overproduction is also considered a cause/consequence common to both apoptosis and necroptosis [25, 26]. Here we show that 15 min of exposure to TSZ was sufficient to significantly increase mitochondrial ROS production in HT29 cells, as detected by the MitoSOX fluorometric assay (Fig. 4B). Likewise, cell incubation with TRZ2 and TRZ7 led to an almost 3-fold increase of mitochondrial ROS, which was partially abolished by Nec-1 and GSK872. Curiously, necroptosis inhibition did not reverse ROS production to the same extent in the case of TRZ7 as it did for TRZ2, suggesting that TRZ7 preferentially triggers apoptosis over necroptosis.

**Fig. 5:**
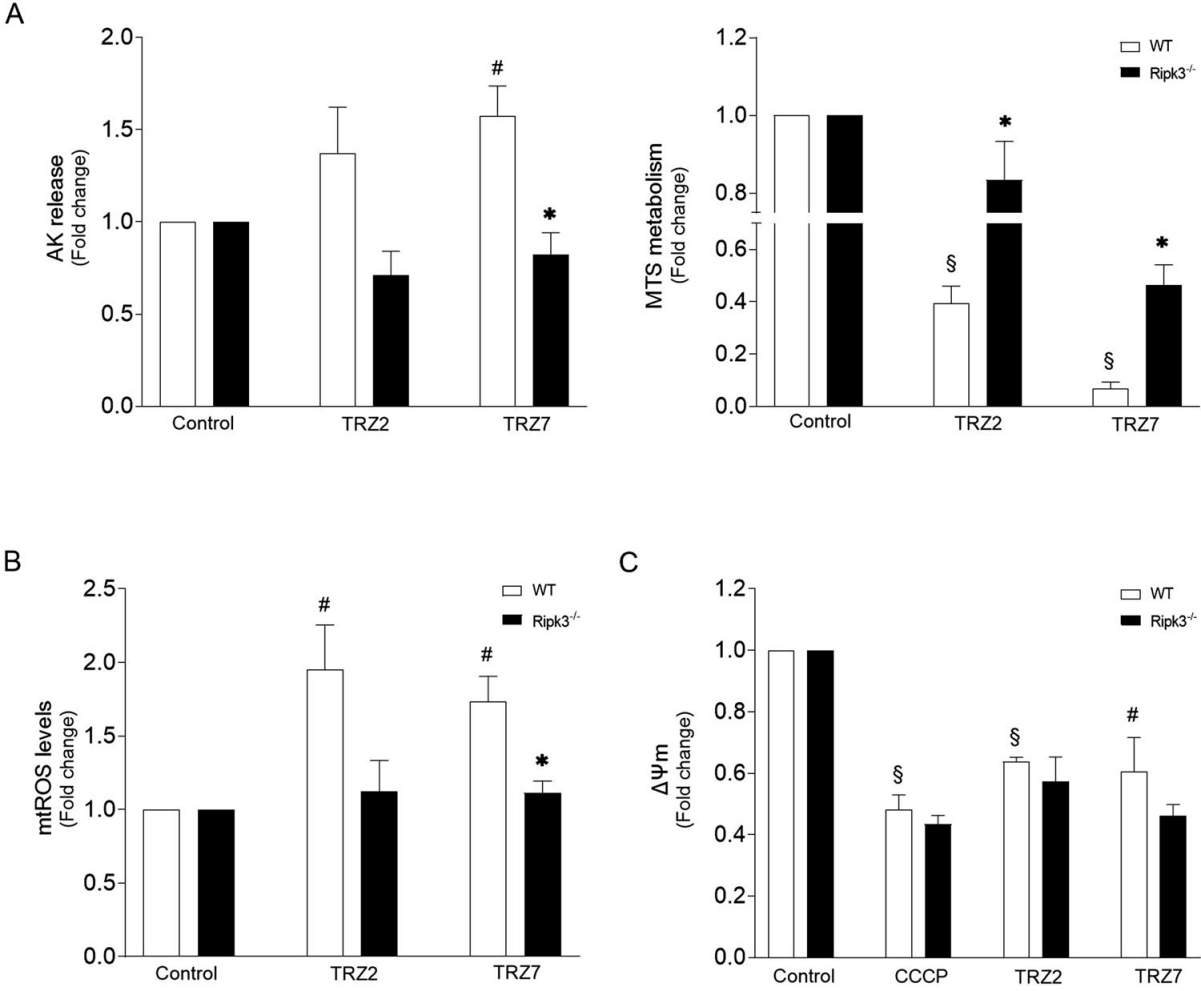
RIPK3 is involved in Ru-TRZ-mediated cell damage. **A** Cell viability was evaluated in AML12 WT and *Ripk3*^*−/−*^ cells after 24 h compound incubation (10 µM), using the AK release and MTS metabolism assays. **B** Mitochondrial ROS production was evaluated after 15 min compound incubation (10 µM) in AML12 WT and *Ripk3*^*−/−*^ cells by fluorometric measurement of MitoSOX Red (580 nm). **C** Mitochondria transmembrane potential (ΔΨ_m_) was evaluated by JC-1 staining of cells exposed to Ru-TRZ (10 µM) for 2 h or CCCP (200 µM) for 4 h (positive control) in AML12 WT and *Ripk3*^*−/−*^ cells. DMSO is the vehicle control. Results are expressed as mean ± SEM fold-change to control, from at least three independent experiments. ^#^*p* < 0.05 and ^§^*p* < 0.001 from control vehicle-treated cells; *p < 0.05 from AML12 WT cells.

Necroptosis execution critically relies on MLKL phosphorylation at Thr357/Ser358 residues (p-MLKL) by RIPK3 kinase, being dependent or independent of RIPK1 kinase activity according to the cellular context [27–30]. Once phosphorylated, MLKL oligomerizes and ultimately induces cell membrane disruption, being an excellent marker of necroptosis commitment [14]. Therefore, we further investigated Ru-TRZ-mediated activation of necroptosis by Western blot analysis. As represented in Fig. 4C, exposure of HT29 cells to Ru-TRZ significantly increased RIPK3 and MLKL phosphorylation compared with DMSO control. Incubation with Nec-1 abolished necroptosis-associated changes. Importantly, when apoptosis was inhibited using Z-VAD, the impact on MLKL activation was massive, with an increase of up to 12-fold in the p-MLKL/MLKL ratio induced by TRZ2, suggesting a shift to necroptosis when apoptosis is compromised. Exacerbation of RIPK1 and RIPK3 phosphorylation levels was also observed. Importantly, incubation with Nec-1 abolished necroptosis-associated changes. As proof of concept, for mechanistic confirmation of necroptosis involvement in Ru-TRZ-induced cell death, we used CRISPR‐Cas9 *Ripk3*‐null immortalized hepatocytes exposed to Ru-TRZ, or vehicle, for 24 h. Comparing to AML12 wild-type, *Ripk3*^*−/−*^ cells were less sensitive to Ru-TRZ exposure as shown by decreased AK release, augmented MTS metabolism (Fig. 5A), and improved mitochondria function (Fig. 5B, C). Overall, these results support our hypothesis that, in addition to apoptosis, Ru-TRZ complexes induce necroptosis.

### Ru-TRZ hybrids act synergistically with conventional chemotherapy drugs and potentiate antiproliferative activity

**Fig. 6:**
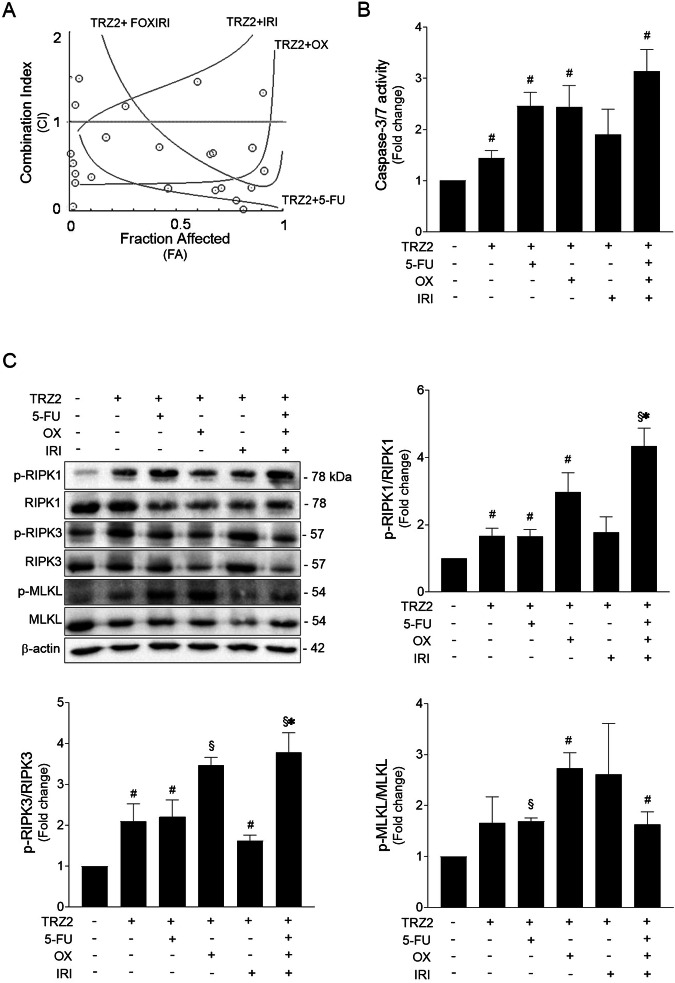
TRZ2 acts synergistically with conventional chemotherapeutics and potentiate cytotoxic effects. **A** Effect of TRZ2 combination with chemotherapeutic drugs 5-FU, oxaliplatin (OX), irinotecan (IRI) or with the triplet (FOXIRI), on HT29 cells, as assessed by median-drug effect analysis method. The CompuSyn generated graph shows the combination index (CI) to the growth-inhibitory effect of each drug (FA). Values below the horizontal line (CI = 1) indicate synergism. **B** Caspase-3/7 activity was determined in HT29 cells treated with TRZ2 alone or in combination (10 µM; 1:1 treatment ratio) for 24 h, using the Caspase-Glo 3/7 assay. **C** Representative immunoblots of necroptosis mediators RIPK1, RIPK3, MLKL, and respective phosphorylated forms in whole-cell extracts from HT29 cells exposed to indicated drug combinations (10 µM; 1:1 treatment ratio) for 24 h (*top left*) and quantifications of p-RIPK1/RIPK1 (*top right*), p-RIPK3/RIPK3 and p-MLKL/MLKL (*bottom*) ratios. Blots were normalized to endogenous β-actin. DMSO was used as a vehicle control. Results are expressed as mean ± SEM fold-change to control, from at least three independent experiments. ^#^*p* < 0.05 and ^§^*p* < 0.001 from control vehicle-treated cells; *p < 0.05 from TRZ2*-*treated cells.

Drug combination is widely used to achieve treatment efficacy in several types of tumors. Regarding colorectal cancer, several combinatory approaches have been approved and demonstrated therapeutic effectivity in patient treatment [31, 32]. Taking this into account, we analyzed the potential of TRZ2 to increase HT29 cell sensitivity to several chemotherapeutics commonly used for the treatment of colon cancer, including 5-fluorouracil (5-FU), oxaliplatin (OX), irinotecan (IRI), and the triplet combination (FOXIRI). First, we determined the IC_50_ value of both individual drugs and combination with TRZ2 in the same proportions (Table 3). Interestingly, the IC_50_ of combination strategies were lower than the IC_50_ of each individual condition, indicating that inclusion of TRZ2 in the treatment regimen markedly increased the antiproliferative effects. Owing to the complexity of the whole-cell biological system, the analysis of drug interactions is not as straight forward, and several mathematical methods may be used to evaluate whether a combination is truly synergistic, additive or antagonistic. The median-drug effect analysis method [33], considers the antiproliferative effect of each drug (FA), the sigmoid shape of the dose-effect curves, and the IC_50_ to calculate a combination index (CI). Notably, as indicated in Table 3, combination of TRZ2 with 5-FU, OX, and FOXIRI resulted in synergistic effects. In contrast, combination with IRI seems to present an antagonistic effect possibly driven by mutually exclusive actions. These results are graphically represented in Fig. 6A, in which values below the horizontal line (CI = 1) indicate synergism. As depicted in Fig. 6B, all regimens tested further increased caspase-3/7 activity in HT29 cells subjected to equal concentration of each drug (10 µM) for 24 h, when compared to TRZ2 alone or vehicle. Overall, these results suggest that combinatory approaches increase cell death.

**Table 3:** IC_50_ of TRZ2, 5-Fluorouracil (5-FU), Oxaliplatin (OX), Irinotecan (IRI), triple combination of 5-FU, OX, and IRI (FOXIRI) and drug combinations of TRZ2 and chemotherapeutics in HT29 cells after 24 h of incubation.

Compound	IC_50_ (μM)	CI	Effect
TRZ2	7.66	-	-
5-FU	29.95	-	-
OX	10.78	-	-
IRI	44.02	-	-
FOXIRI	12.80	-	-
TRZ2 + 5-FU	4.11	0.12	Synergistic
TRZ2 + OX	2.72	0.60	Synergistic
TRZ2 + IRI	6.75	1.89	Antagonistic
TRZ2 + FOXIRI	4.31	0.49	Synergistic

Table also displays the Combinatory Index (CI) values and associated effects (Synergistic or Antagonistic). CI < 0.8 depicts synergism between drugs, 0.8 < CI < 1.2 depicts additivity and CI > 1.2 depicts antagonism.

Finally, western blot analysis of protein samples extracted from HT29 cells treated with the aforementioned drug combinations revealed a significant activation of necroptosis mediators in HT29 cells, not always higher than observed when TRZ2 is alone, but sufficient to suggest that necroptotic cell death is also prominent. Importantly, combination of TRZ2 with oxaliplatin and with the three drugs showed a more pronounced increase in necroptosis mediators than TRZ2 alone (Fig. 6C).

## Discussion

We report on a newly synthesized series of Ru triazene hybrids that are selective and highly cytotoxic for HCT116 and HT29 colon cancer cells. Our uptake studies demonstrate that Ru-TRZ complexes accumulate in cancer cells. We confirmed the presence of Ru in whole-cell lysates with preferential accumulation in membrane organelles. The fundamental role of Ru(II) structural moiety during the uptake process was confirmed by confocal microscopy, considering that fluorescently-tagged TRZs are absorbed, but corresponding organic ligands are not. Compound-induced cell damage is also dependent of Ru presence, thus validating selectivity against tumor cells. We also showed that Ru-TRZ are specifically targeted to mitochondria as previously reported for other Ru compounds [11, 12, 21]. The mechanistic underpinning of these Ru compounds involves the uptake of cationic lipophilic compounds by mitochondria with a negative potential. In cancer cells, the potential is lower than in normal cells, leading to a selective absorption process.

Here we show that Ru-TRZ hybrids impact on mitochondria function, leading to cell death, characterized by caspase activation, apoptosis, and necroptosis. Proof-of-concept in vivo experiments suggests that treatment with a Ru-TRZ hybrid decreases tumor burden in mice.

The exact role of necroptosis during cancer development remains to be fully elucidated. Due to its proinflammatory profile, necroptosis promotes tumor progression and metastasis [34]. In contrast, an antitumoral function of necroptosis is supported by the downregulation of necroptotic mediators and correlation with reduced overall survival of patients with different types of cancer, including colorectal cancer [35–37]. Moreover, since the apoptotic machinery is often compromised by oncogenic signals leading to treatment resistance, activation of necroptosis in cancer cells may be exploited as an effective approach to circumvent apoptotic resistance.

A growing number of compounds have been described with the ability to trigger necroptosis in cancer cells [10, 34]. However, metal-based triggers have rarely been reported. Here we prove that, in addition to apoptosis, Ru-TRZ induces cell death by necroptosis. At low doses, these complexes are highly cytotoxic for cancer cells inducing a significant loss of cell viability that is partially decreased by Z-VAD (apoptosis inhibitor), but also by Nec-1 (RIPK1 inhibitor) and GSK872 (RIPK3 inhibitor), suggesting that both cell death routines are activated. Activation of necroptosis mediators RIPK1, RIPK3 and MLKL upon treatment with Ru-TRZ and the inhibitory effects after co-incubation with Nec-1 further suggest necroptosis activation. Remarkably, when apoptosis is inhibited, the phosphorylation levels of necroptosis mediators are exacerbated, implying a shift from a dual apoptotic/necroptotic cell death to a mainly necroptotic approach. In fact, Z-VAD-induced caspase-independent cell necroptosis was already reported in several other studies [38, 39]. As an example, addition of Z-VAD to lipopolysaccharide (LPS), polyinosinic-polycytidylic acid (poly I:C), TNF-α or BV6 may sensitize cells to compound-mediated citotoxicity by potentiating necroptosis, depending on the cell type and context [40, 41]. Of note, although Nec-1 has been identified as a specific inhibitor of RIP1 kinase activity, preventing RIPK1 autophosphorylation and necrosome assembly, Nec-1 off-target effects and lack of specificity have been largely described [42]. In the present study, the less pronounced loss in cellular viability and reduced mitochondrial damage obtained in cells with genetic ablation of RIPK3, strongly corroborates that Ru-TRZ hybrids induce necroptotic cell death.

Regarding RIPK3 inhibitors, it has been shown that inhibition of RIPK3-dependent necroptosis, may trigger caspase activation and apoptotic cell death, independently of necroptosis machinery [43]. In fact, here we also observed that addition of GSK872 to Ru-TRZ-treated L929 cells does not change the levels of cell death, suggesting a shift of necroptosis to apoptosis.

In this study, we also explore the potential benefit of introducing Ru-TRZ in combination therapy regimens. Drug combination is widely used to achieve treatment efficacy of several types of tumors. In respect to colon cancer treatment, combinations between metal, non-metal compounds, targeted drugs and immunotherapy approaches have been made and have shown to improve significantly patient treatment outcomes [44, 45].The main goal is to achieve a synergistic therapeutic effect accompanied by decreased toxicity, and diminished drug resistance [46]. The definition of synergism is that the combination of drugs is more effective than each drug separately, meaning that one of the agents augments the action of the second. Antagonism characterizes a combination that is less effective than the single agents, when one of the agents counteracts the actions of the other. Antagonistic action may be desired when toxicity is a concern, and one drug decreases the side effects of another drug [47].

Here, we show that Ru-TRZ combination with chemotherapeutic drugs commonly used in the treatment of colon cancer [31, 48], 5-FU, oxaliplatin and irinotecan, potentiates treatment efficacy, considering the increased cytotoxic effects induced in HT29 cells by combination drugs comparing with individual drugs. Combinations of TRZ2 with both 5-FU or oxaliplatin or the three chemotherapeutic drugs have a synergistic effect, with hyperactivation of apoptosis. Considering the activation of necroptosis mediators, the combination of TRZ2 with oxaliplatin displays consistent increase in the activation of RIPK1,3 and MLKL mediators comparing with TRZ2 alone. The combination of TRZ2 with the three drugs, also impacts in the activation of RIPK1 and RIPK3 comparing with TRZ2 alone. The fact that this is not observed for MLKL, together with the huge increase in caspase activity, may suggest that the triple combination favors apoptosis, both dependent and independent of RIPK1 activity. TRZ2 plus 5-FU combination also seems to favor apoptosis. Regarding irinotecan, although combination with TRZ2 potentiate its activity considering lower IC_50_ values, the combinatory effect is antagonist, probably due to irinotecan lower potency. This antagonism is observed in caspase activity and necroptosis mediators, with no significant benefits for combination.

In conclusion, we identified a new family of Ru-TRZ hybrids that are able to induce cell death by apoptosis and necroptosis. The activation of necroptosis is exacerbated when apoptosis is blocked, which is particularly relevant in scenarios of apoptosis tumor resistance. The mechanism behind Ru-TRZ-induced cell death appears to be mitochondria-dependent with the activation of necroptosis machinery depending on RIPK3 and MLKL proteins for full activity. Overall, Ru-TRZ might be used as alternative chemotherapeutic agent when the cellular apoptotic machinery is compromised. In addition, by improving antiproliferative activity and cell death induced by conventional drugs, combination strategies with Ru-TRZ could counteract drug resistance phenomena.

## Materials and methods

### Chemical synthesis

Reagents were from commercial sources and used without further purification. ^1^H and ^13^C nuclear magnetic resonance (NMR) spectra were recorded on a Bruker Ultra-Shield 300 MHz spectrometer operating at 300 MHz, at probe temperature. Chemical shifts are quoted on the δ scale, in parts per million, using residual solvent peaks as the internal standard. Coupling constants (*J*) are reported in hertz with the following splitting abbreviations: s, singlet; br, broad; d, doublet; t, triplet; q, quartet; p, quintet; m, multiplet. Thin layer chromatography used 60 F254 silica gel. Flash column chromatography was performed using Merck 60 (230–400 mesh ASTM) silica gel. Ultraviolet (UV) spectra were traced in a Thermo Scientific Evolution 201 UV−visible spectrophotometer, and fluorescence measurements taken on a SHIMADZU RF-6000 instrument.

Detailed procedures for the synthesis of triazene, amide, and NBD-labeled ligands [49] are presented in Supplementary Methods, and NMR spectra added as Supplementary Figures (Figs. S1–11). Synthesis of TRZ1: to a Schlenk tube charged with 1-(4-cyanophenyl)-3-methyltriazene (0.25 mmol) and THF (5 mL), NaH (0.3 mmol) was added. After 1 h stirring, the mixture was transferred via cannula to a second Schlenk tube charged with [(η^5^-C_5_H_5_)RuCl(PPh_3_)_2_] (0.2 mmol), AgCF_3_SO_3_ (0.2 mmol) and THF (5 mL), and previously stirred for 1 h at room temperature. The mixture was then stirred overnight at room temperature, filtered, and pumped to dryness. The crude product was purified by column chromatography (DCM) affording the pure product as a dark orange solid (yield 37%). High-Resolution Mass Spectrometry (HRMS): C_31_H_27_N_4_PRu, theoretical *m/z* 611.0933 [M+Na]^+^, experimental *m/z* 611.0955. During synthesis of compounds TRZ2 to TRZ8, TRZ2-PEG, TRZ2-PEG-Fluo, and TRZ7-Fluo, to a Schlenk tube charged with [(η^5^-C_5_H_5_)RuCl(Dppe)] (0.2 mmol), AgCF_3_SO_3_ (0.2 mmol) and appropriate ligands (0.21 mmol) was added DCM (10 mL). After stirring overnight at room temperature, solutions were filtered to remove AgCl precipitate and pumped to dryness. Crude solids were washed with n-hexane (2 × 10 mL), dried, and next recrystalized by slow diffusion of n-hexane in DCM (or acetone for TRZ2) solutions of the compounds. TRZ2: red; yield 68%.^1^H NMR (300 MHz, DMSO-D6). HRMS: *m/z* 795.1990 (+3.5 ppm). TRZ3: pale yellow; yield 76%.^1^H NMR (300 MHz, DMSO-D6). HRMS: *m/z* 683.1322 (−0.3 ppm). TRZ4: yellow; yield 79%.^1^H NMR (300 MHz, DMSO-D6). HRMS: *m/z* 829.1838 (+3.8 ppm). TRZ5: orange; yield 64%.^1^H NMR (300 MHz, DMSO-D6). HRMS: *m/z* 739.1721 (+3.0 ppm). TRZ6: yellow; yield 55%.^1^H NMR (300 MHz, DMSO-D6). HRMS: *m/z* 886.2503 (+2.1 ppm). TRZ7: yellow; yield 87%.^1^H NMR (300 MHz, DMSO-D6). HRMS: *m/z* 753.1743 (+0.9 ppm). TRZ2-PEG: yellow; yield 65%.^1^H NMR (300 MHz, DMSO-D6). HRMS: *m/z* 940.2537 (+9.2 ppm). TRZ2-PEG-Fluo: dark orange; yield 57%.^1^H NMR (300 MHz, DMSO-D6). HRMS: *m/z* 1158.2917 (+2.1 ppm). TRZ8: yellow; yield 49%.^1^H NMR (300 MHz, DMSO-D6). HRMS: *m/z* 898.2243 (+1.2 ppm). TRZ7-Fluo: dark orange; yield 76%.^1^H NMR (300 MHz, DMSO-D6). HRMS: *m/z* 1116.2623 (−4.6 ppm).

### X-ray diffraction studies

Single crystal X-ray diffraction experiments of TRZ2, TRZ5, and TRZ7 were performed with a Bruker X8APEX area detector diffractometer with graphite-monochromated Mo Kα (*λ* = 0.71073 Å) radiation. The crystals were mounted on a Kaptan loop with a protectant oil (Fomblin) to prevent air diffusion to the crystal and reduce exposure. Data were collected with the X-ray generator at 50 kV and 30 mA and monitored with APEX3 program. An empirical absorption correction was enforced using Bruker SADABS (Bruker Analytical Systems, Madison, WI, USA) and data reduction was done with Bruker SAINT program (SAINT+, release 6.22; Bruker Analytical Systems, Madison, WI). Structure was solved with Bruker SHELXS [50] and refined by full-matrix-least-squares on F^2^ using SHELXL [20] programs within WINGX-Version 2020.2 [51]. Non-hydrogen atoms were refined with anisotropic thermal parameters. Hydrogen atoms were placed in calculated positions and allowed to refine in the parent carbon atoms. Mercury 2020.3.0 was used for molecular representations [52]. PLATON was used for the determination of hydrogen bond interactions [53]. A summary of the crystal data, structure solution, and refinement parameters is given in Supplementary Table S1.

### Cell culture and treatments

The L929 murine fibrosarcoma and the HT29 human colorectal adenocarcinoma cell lines were originally obtained from ATCC Collections. HCT116 human colorectal adenocarcinoma and HepG2 human liver cancer cells were from ECACC Culture Collection and AML12 murine hepatocytes from LGC Standards. AML12 *RIPK3*^*−/−*^ cells were obtained using CRISPR/Cas9 technologies and kindly provided by Dr. Jérémie Gautheron (Sorbonne Université, Inserm, Centre de Recherche Saint‐Antoine, Paris, France). HepG2 and L929 cells were grown in DMEM, whereas HT29 cells were grown in RPMI 1640 and HCT116 cells in McCoy’s 5 A. Cell media were supplemented with 10% heat-inactivated fetal bovine serum (FBS) and 1% antibiotic/antimycotic solution. AML12 wild-type and *RIPK3*^*−/−*^ cells were grown in DMEM-F12 supplemented with 10% FBS, 1% antibiotic/antimycotic, 1% insulin-transferrin-selenium (ITS), and 40 ng/ml of dexamethasone (Sigma-Aldrich, St. Louis, MO, USA). Cell culture media and supplements were acquired from Gibco (Thermo Fisher Scientific, Waltham, MA, USA). All cell lines were maintained at 37°C under a humidified atmosphere of 5% CO_2_.

As positive control for necroptosis induction, HT29 cells were treated with 20 ng/mL recombinant human TNF-α (#300-01A, PeproTech EC Ltd., London, UK), 10 μM Z-Val-Ala-Asp-fluoromethylketon (Z-VAD-FMK or Z-VAD) pan-caspase inhibitor (#ALX-260-020, Enzo Life Sciences, Farmingdale, NY, USA), and 250 nM BV6 Smac mimetic (#B4653, ApexBio, Houston, USA), L929 cells were treated with 10 ng/mL recombinant murine TNF-α (#315-01 A, PeproTech), and HepG2 cells were treated with 10 μM doxorubicin (DOXO) (#D1515, Sigma-Aldrich). Necrostatin-1 (Nec-1), a RIPK1 inhibitor (30 μM; #N9037, Sigma-Aldrich), and GSK872, a RIPK3 inhibitor (10 μM; HY-101872, MedChemExpress), were used as necroptosis inhibitors to confirm loss of cell viability by a necroptotic stimulus. To block apoptosis, cells were incubated with 10 μM Z-VAD. Dimethyl sulfoxide (DMSO; Sigma-Aldrich) was the vehicle control (0,001%, v/v). Sample size was estimated based on previous experience and is indicated in figure legends. Investigators were blinded to the group allocation when assessing the outcome.

### Cell death assessments

For evaluation of cell death and viability, cells were plated in 96-well plates at 5 × 10^3^ cells/well for next-day treatments. MTS metabolism was evaluated as an indicator of cell viability using CellTiter 96^®^ AQ_ueous_ non-radioactive cell proliferation assay (Promega, Madison, WI, USA), according to the manufacturer’s instructions. Changes in absorbance were measured at 490 nm using a GloMax^®^-MultiDetection System (Promega). General cell death was evaluated using the ToxiLight^TM^ BioAssay Kit (Lonza Walkersville Inc., Walkersville, MD, USA), by determining the quantities of adenylate kinase (AK) released from cells with permeabilized plasma membranes and present in the extracellular medium. The bioluminescent signals were recorded using also the GloMax^®^-MultiDetection System.

To determine caspase-3/7 activity, HT29 cells were seeded in 96-well plates at 5 ×10^3^ cells/well. Twenty-four hours later, cells were incubated with 10 µM Ru-TRZ, or vehicle control, for additional 24 h. Caspase-3 and -7 activation status was measured using the Caspase-Glo^®^ 3/7 Assay (Promega), according to the manufacturer’s instructions. The luminescent signal was recorded using the GloMax^®^-MultiDetection System (Promega).

Apoptotic cell death was quantified by flow cytometry using the Guava Nexin Reagent kit (Luminex, Texas, USA). HT29 cells were plated in 24-well plates at 3 × 10^4^ cells/well. Twenty-four hours later, cells were exposed to 10 µM Ru-TRZ or vehicle control for additional 24 h. Briefly, cell supernatant and adherent cells were collected, centrifuged at 500 *g* for 5 min and resuspended in PBS containing 2% FBS. Next, equal volumes of the cell suspension and the Guava Nexin reagent were mixed, incubated for 20 min protected from light, and assayed promptly, using a Guava easyCyte 5HT flow cytometer (Luminex). Sample acquisition and analysis were performed using the InCyte software module.

### Drug screening

The primary drug screen was performed using the HCT116 and HT29 cell lines. Cells were seeded in 96-well plates at 5 × 10^3^ cells/well and 24 h after, exposed to Ru-TRZ compounds and derivatives at a range of concentrations from 0.012 to 243 μM. Cell proliferation was assessed 72 h later using the MTS assay. As a secondary screen, selected hits were tested using HT29, HepG2, and AML12 wild-type cells seeded in 96-well plates at 5 × 10^3^ cells/well. In the following day, cells were exposed to selected compounds at the same range of concentrations for additional 24 h. Cell death was evaluated by the ToxiLight assay. All measurements were performed in duplicate. The half-maximal effective concentration (EC_50_) and half-maximal inhibitory concentration (IC_50_) were calculated using the GraphPad Prism Software version 8.0.2 (GraphPad Software, Inc., San Diego, CA, USA) with the log (inhibitor) versus response (variable slope) function.

### Animal studies, injections, and monitoring

Male nude mice (8–10 weeks old) were purchased from Charles River (Barcelona, Spain). Animals were kept in individually ventilated cages, under strict hygiene conditions, on a 12 h light/12 h dark cycle, at 20–24 ^o^C and 50–65% humidity. Mice had free access to sterilized diet and sterilized acidified water. All animal experiments were conducted according to the institutional Animal Welfare Body (ORBEA, Faculty of Pharmacy, Universidade de Lisboa, approved by the competent national authority Direção-Geral de Alimentação e Veterinária DGAV), EU Directive (2010/63/UE) and Portuguese laws (DR 113/2013, 2880/ 2015, 260/2016 and 1/2019) for the use and care of animals in research.

For xenograft tumor induction, a total of 1 × 10^6^ HT29 cells were suspended in 100 μL of PBS and injected subcutaneously in the right flank of nude (athymic) male mice [54]. When tumors reached a volume of around 200 mm^3^, treatment schedule was initiated. Male C57BL/6 mice 8–10 weeks old were purchased from Charles River (Barcelona, Spain) and randomly divided in 2 groups of 5 animals each. No statistical methods were used to determine sample size estimate in this pilot study. The test group received 5 mg/kg TRZ2 dissolved in 1% DMSO by intravenous route, in a total of ten administrations for 2 weeks, while the control group received the vehicle (1% DMSO in PBS). Mice were monitored every day for pain or distress and body weight was registered. Tumor size was measured over the treatment protocol using a digital calliper and volumes were calculated according to the formula: *V* (mm^3^) = (*L* × *W*^2^)/2, where *L* and *W* represent the longest and shortest axis of the tumor, respectively [54].Two days after the final administration, mice were sacrificed, and primary tumors were excised and weighed. Investigators were blinded to the group allocation when assessing the outcome.

### Cell fractionation and subcellular ruthenium

HT29 cells were seeded in 100 mm culture dishes at 2 × 10^6^ cells/dish. In the next day, cells were incubated with 5 µM Ru-TRZ for additional 24 h. At this time-point, cells were collected by trypsinization, centrifuged at 500 *g* for 5 min, and washed twice with ice-cold PBS. Finally, cell pellets were resuspended in PBS for whole-cell acid digestion or subcellular fractionation. The FractionPREP™ Cell Fractionation kit (Biovision, Abcam, Cambridge, UK) was used to extract four subcellular protein fractions (cytosolic, nuclear, membrane/particulate, and cytoskeletal), according to the manufacturer’s protocol. To quantify the amount of intracellular Ru, an acid digestion was performed by treating whole-cell pellets or subcellular fractions with 500 µl of a mixture of acids containing 35% HNO_3_ and 65% HCl, in a glass tube and during 1 h, under agitation, in a 90°C water bath. After cooling, samples were treated with 500 µL H_2_O_2_ and incubated during 1 h, under agitation at 90°C. Digested samples were transferred to a clean 2 mL microtube, and the glass digestion tube was washed with 400 µL 1 M HCl. The washing content was transferred to the microtube containing the digested sample. Final samples were analyzed by inductively coupled plasma-mass spectrometry (ICP-MS) to determine the amount of Ru that was uptaken by cells.

Molecular analyses were performed using an ACQUITY ultra-performance liquid chromatography system (Waters, Mildford, MA, USA) coupled to a hybrid quadrupole orthogonal acceleration time-of-flight mass spectrometer Xevo G2 (Waters Corp, Manchester, UK). Data were acquired from *m/z* 50 to 1200 in data-independent acquisition mode to obtain information about the protonated molecule and adducts if present (*e.g*., sodium adducts) and fragment ions in a single run. MS data were acquired and processed using MassLynx data station operation software version 4.1 (Waters). Quantitative determination of Ru in the HNO_3_-digested samples was conducted with an iCAP-RQ ICP-MS instrument (Thermo Fisher Scientific, Bremen, Germany). A MicroFlow PFA ST nebulizer (Elemental Scientific, Omaha, NE, USA) and a double-pass quartz cyclonic spray chamber cooled to 2.7 °C were used. The generated aerosol was transferred into the plasma via a 2.5 mm quartz injector. Nickel Sampler and skimmer cones and a “high matrix” skimmer insert was mounted. All measurements were performed in collision mode using helium gas flow to improve sensitivity and to avoid polyatomic interferences. Samples were suitably diluted with double deionized water and nebulized into the ICP-MS. Signal at m/z 101, 102 was monitored. Concentration mean values from both m/z were obtained. An 8-point external calibration curve, from 0 to 25 ng mL^−1^, were used for the quantification. Each calibrating standard was freshly prepared by subsequent dilution from a certified Ru standard stock solution: VHG-PRUH-100, 1000 mg L^−1^ Ru in 20% HCl (LGC Standards, Barcelona, Spain). Reagents free of Ru were checked from the analysis of blank solutions with 1% HNO_3_.

### Cellular uptake

HT29 cells were seeded on µ-Slide 8 well glass bottom (ibidi, Fitchburg, WI, USA) at 3 × 10^4^ cells/well and cultured for 24–36 h before each experiment. Fluorescently labeled compounds TRZ2-PEG-Fluo and TRZ7-Fluo, and their modified versions without Ru atom, or vehicle control, were added to cells at 1 µM in Dulbecco’s phosphate-buffered saline (DPBS) (Thermo Fisher Scientific) for 15 min. Then, a washing step with DPBS was included and cells were subsequently labeled with MitoTracker™ Deep Red and Hoechst 33342 dyes (both from Thermo Fisher Scientific), according to supplier’s instructions. Unbounded dyes were removed by washing with DPBS. Cells were then imaged using a laser scanning confocal microscope (Leica TCS-SP5; Leica, Germany) and a two-photon–excited fluorescence microscope (Ti:sapphire laser; Spectra-Physics Mai Tai BB). Images were collected with 512 × 512 pixels at a scan rate of 100 Hz with the 488 nm Ar+ laser line for fluorescent compounds, the 633 nm He-Ne laser line (for MitoTracker™ Deep Red; Thermo Fisher Scientific), and the 780 nm with the Ti:sapphire laser (for Hoechst 33342). A 63 × 1.2 N.A. water immersion objective was used (HCX PL APO CS 63.0 × 1.20 WATER UV; Leica).

### Mitochondrial transmembrane potential

JC-1 (Sigma-Aldrich) is a cationic dye that represents a reliable fluorescent probe for the assessment of mitochondrial transmembrane potential (ΔΨm). JC-1 exhibits potential-dependent accumulation in mitochondria, indicated by a fluorescence emission shift from green (~530 nm), corresponding to JC-1 monomers and indicative of low membrane potential, to red (~590 nm), corresponding to JC1 aggregates and reflecting higher mitochondrial potential. HT29 or AML12 cells were plated on a 96-well plate at 5 × 10^3^ cells/well and on the next day treated with Ru-TRZ compounds (1 and 5 µM) for 2 h. Carbonyl cyanide m-chlorophenyl hydrazone (CCCP), a mitochondrial depolarizing agent, was used as positive control (200 µM; 4 h). Emitted fluorescence was measured at 530 nm and 590 nm using the GloMax^®^-MultiDetection System (Promega) and the ratio 590/530 emission intensity was calculated.

### Mitochondrial ROS production

Mitochondrial superoxide abundance was quantified by fluorometric quantification. Briefly, HT29 or AML12 cells were plated on a 96-well plate at 5 ×10^3^ cells/well and on the next day treated with 10 µM Ru-TRZ, or vehicle control, for additional 15 min. MitoSOX™ Red (Invitrogen, Thermo Fisher Scientific) diluted 1:1000 in HBSS was added (100 μL per well) and incubated at 37 °C in a CO_2_ incubator for 10 min. Cells were then washed with PBS to remove the excess of dye and fluorescence intensity was measured using a Varioskan™ LUX multimode microplate reader (Thermo Scientific) with excitation and emission wavelengths of 510 and 580 nm, respectively. Fluorescence values were normalized by the cell mass content.

### Steady state protein production

For isolation of total protein extracts, HT29 cells were seeded in 60 mm dishes at 4 × 10^5^ cells/dish. In the following day, cells were incubated with 10 µM Ru-TRZ, in the presence or absence of Nec-1 or Z-VAD, for additional 24 h. Fifty μg of total protein extracts were separated on 8% sodium dodecyl sulfate-polyacrylamide electrophoresis gels (SDS-PAGE), transferred onto nitrocellulose membranes (RTA Transfer Kit; Bio-Rad, Hercules, CA, USA) and blocked with 5% (w/v) non-fat dry milk in TBS-Tween-20 (0.5% v/v). Blots were incubated overnight at 4°C with primary rabbit antibodies reactive to p-MLKL (1:1000; ab187091; Abcam), MLKL (1:1000; ab183770; Abcam), p-RIPK3 (1:1000; D6W2T; Cell Signaling, Danvers, MA, USA), RIPK3 (1:1000; E1Z1D; Cell Signaling), p-RIPK1 (1:1000; D8I3A; Cell Signaling), RIPK1 (1:1000; D94C12; Cell Signaling) and PARP-1 (1:2000; sc-7150; Santa Cruz Biotech, Dallas, TX, USA). Following incubation with secondary antibody conjugated with horseradish peroxidase (1:5000, Bio-Rad) for 2 h at room temperature, membranes were processed for protein detection by chemiluminescence using SuperSignal^TM^ West Femto Maximum Sensitivity Substrate (Pierce, Thermo Fisher Scientific) or Immobilon Western Chemiluminescent HRP Substrate (Millipore, Burlington, MA, USA), on a ChemiDoc XRS-imaging system (Bio-Rad). β-actin (1:40,000, A5541; Sigma-Aldrich) was used as loading control. Relative intensities of protein electrophoretic bands were analyzed using the ImageLab^TM^ densitometric analysis software (version 6.0.1; Bio-Rad). Full and uncropped Western blots were uploaded as Supplementary Material (Figs. S12–14).

### Drug combination index

In drug combination studies, cells were incubated with TRZ2 combined with 5-fluorouracil (5-FU), oxaliplatin (OXA), and irinotecan (IRI) chemotherapeutics (kindly provided by Hospital Santa Maria, Lisbon, Portugal) at a ratio of 1:1. The effect of combined treatments was evaluated using the CompuSyn software 1.0 (ComboSyn, Inc. and PD Science, LLC). Briefly, the combination index (CI) is a tool to analyze the interaction between drugs allowing to distinguish between synergistic, additive, and antagonistic effects. CI values are calculated considering the concentration of drugs in combination to produce a specific effect (Fraction affected—Fa) and the concentration of drugs to produce that same effect individually. The final CI value is calculated by the average of CI values at Fa 50, 75, 90, and 95%. CI value < 1 indicates synergism while a CI value > 1 indicates antagonism. Fa-CI plot between the levels of different fractions affected against the CI values was also created using CompuSyn software to interpret the type of interactions between TRZ2 and chemotherapeutic drugs. The data points below the additivity line (CI = 1) indicate synergism.

### Supplementary information

Supplementary material

## Data Availability

The authors confirm that the data supporting the findings of this study are available within the article or in supplementary materials.
