# Supplementary material for: Necroptosis induced by ruthenium (II) complexes as mitochondrial disruptors
Source: Cell Death Discov. 2024 May 28;10:261. doi: 10.1038/s41420-024-02033-z (PMC11133381; doi:10.1038/s41420-024-02033-z)
Supplement: Supplementary file 1 — Supplementary material [file 41420_2024_2033_MOESM1_ESM.docx]

**Supplementary Information**

**Necroptosis Induced by Ruthenium (II) Complexes as Mitochondrial Disruptors**

Joana Gonçalves^1^, Joana D. Amaral^1^, Rita Capela^1^, Maria de Jesus Perry^1^, Cláudia Braga^1^, Maria Manuela Gaspar^1,2^, Fátima M. Piedade^3,4^, Lubertus Bijlsma^5^, Antoni Roig^5^, Sandra N. Pinto^6^, Rui Moreira^1^, Pedro Florindo^1^, Cecília M. P. Rodrigues^1^

^1^Research Institute for Medicines (iMed.ULisboa), Faculty of Pharmacy, Universidade de Lisboa, Lisbon, Portugal

^2^Instituto de Biofísica e Engenharia Biomédica, Faculty of Sciences, Universidade de Lisboa, Lisbon, Portugal

^3^Departamento de Química e Bioquímica, Faculty of Sciences, Universidade de Lisboa, Lisbon, Portugal

^4^Centro de Química Estrutural, Institute of Molecular Sciences, Instituto Superior Técnico, Universidade de Lisboa, Lisbon, Portugal

^5^Environmental and Public Health Analytical Chemistry, Research Institute for Pesticides and Water, University Jaume I, Castelló, Spain

^6^iBB-Institute for Bioengineering and Biosciences, Department of Bioengineering, Instituto Superior Técnico, Universidade de Lisboa, Lisbon, Portugal

Correspondence: Cecilia Rodrigues, Ph.D., iMed.ULisboa, Faculty of Pharmacy, Universidade de Lisboa, Av. Prof. Gama Pinto, 1649-003 Lisbon, Portugal, cmprodrigues@ff.ulisboa.pt, Tel.: +351 21 794 6490

**Supplementary Methods**

**Chemical synthesis of ligands**

**Synthesis of triazene ligands**

1-(4-cyanophenyl)-3-methyltriazene (L1) was synthesized according to previously reported procedure (50). Triazene ligand L2 was synthesized according to previously reported procedure (20), and L2-PEG was synthesized following an analogous procedure from 1-azido-3,6,9-trioxaundecanoic acid.

**Synthesis of amide ligands**

4-cyanoaniline (0.5 mmol) was dissolved in DMF (5 mL) and added to a solution of TBTU (0.5 mmol), DIPEA (0.8 mmol), and the appropriate carboxylic acid (L6: 1-(9H-fluoren-9-yl)-3-oxo-2,7,10,13-tetraoxa-4-azahexadecan-16-oic acid; L7: butyric acid; L8: 1-azido-3,6,9-trioxaundecanoic acid; 0.5 mmol) in DMF (5 mL). The mixture was stirred overnight at room temperature; for L6, piperidine was added to the mixture and stirred for further 30 min for terminal primary amine deprotection (Fmoc). The volatiles were then removed under reduced pressure, and the crude products were purified by flash chromatography (AcOEt:n-hexane mixtures), affording the pure ligands.

**Synthesis of NBD-labelled ligands**

To a mixture of CuI (0.5 mmol), azide (L2-PEG, L8; 0.5 mmol) and DIPEA (1 mmol) in THF (5 mL) was added N-(3-azidopropyl)-7-nitrobenzo[c][1,2,5]-oxadiazol-4-amine (51) (0.5 mmol), and the mixture was stirred at 75 °C for 3 h. Solvent was then removed under reduced pressure, and the crude products were purified by flash chromatography (eluent of 0−8% MeOH in DCM), affording ligands L2-PEG-Fluo and L7-Fluo.

**Synthesis of LTRZ4, LTRZ5 e LTRZ7**

**3-benzoyl-1-(4-cyanophenyl)-3-methyltriazene (LTRZ4)**

1-(4-cyanophenyl)-3-methyltriazene (1.2 mmol) was dissolved in THF (2 mL) and NaH (80% dispersion in mineral oil, 0.6 mmol) was added. Then, pyridine (1.4 mmol) was added into the reaction mixture. After 30 min, benzoyl chloride (1.4 mmol) was added and the reaction mixture was left to stirr overnight. After completion, the reaction mixture was evaporated to dryness and the residue was purified by column chromatography (1:1 – Hex: EtOAc) to give the pure product as a pale yellow solid (41% yield). ^1^H NMR (300 MHz, CDCl_3_): δ 7.69-7.64 (m, 4H), 7.55-7.43 (m, 5H), 3.65 (s, 3H). ^13^C NMR (75 MHz, CDCl_3_) δ 172.06, 151.61, 134.08, 133.38, 131.59, 130.08, 127.98, 123.00, 118.56, 112.21, 29.18.

**1-(4-cyanophenyl)-3-dimethyltriazene (LTRZ5)**

The compound **LTRZ5** was synthesized according to procedure published in reference (52). Pale yellow solid (89% yield). ^1^H NMR (300 MHz, CDCl_3_): δ 7.57 (d, *J* = 8.7 Hz, 2H), 7.45 (d, *J* = 8.7 Hz, 2H), 3.54 (s, 3H), 3.23 (s, 3H). ^13^C NMR (75 MHz, CDCl_3_) δ 154.31, 133.07, 121.07, 119.64, 107.81, 43.47, 36.20. HRMS: *m/z* 175.0988 (+2.3 ppm).

**L6:** 3-(2-(2-(2-aminoethoxy)ethoxy)ethoxy)-N-(4-cyanophenyl)propanamide

No NMR or HRMS data, just for the complex TRZ6.

**L7: N-(4-cyanophenyl)butanamide (LTRZ7)**

Yellow solid (yield = 87%). ^1^H NMR (300 MHz, CDCl_3_) δ 8.09 (s, 1H), 7.70 (d, *J* = 8.8 Hz, 2H), 7.57 (d, *J* = 8.7 Hz, 2H), 2.37 (t, *J* = 7.4 Hz, 2H), 1.82 – 1.64 (sext, *J* = 7.4 Hz, 2H), 0.98 (t, *J* = 7.4 Hz, 3H). ^13^C NMR (75 MHz, CDCl_3_) δ 172.29, 142.53, 133.29, 119.67, 119.10, 106.61, 39.64, 18.96, 13.77. HRMS: *m/z* 189.1034 (+3.2 ppm).

**L8**: 2-(2-(2-(2-azidoethoxy)ethoxy)ethoxy)-N-(4-cyanophenyl)acetamide

No NMR data. HRMS [M+Na]^+^: *m/z* 356.1337 (+0.6 ppm)

**L2-PEG:**(E)-4-(3-(2-(2-(2-(2-azidoethoxy)ethoxy)ethoxy)acetyl)-3-methyltriaz-1-en-1-yl)benzonitrile

No NMR. HRMS [M+Na]^+^: *m/z* 398.1564 (+1.5 ppm).

**L2-PEG-Fluo:**(E)-4-(3-methyl-3-(2-(2-(2-(2-(4-(((7-nitrobenzo[c][1,2,5]oxadiazol-4-yl)amino)methyl)-1H-1,2,3-triazol-1-yl)ethoxy)ethoxy)ethoxy)acetyl)triaz-1-en-1-yl)benzonitrile

Orange solid (yield = 65%). ^1^H NMR (300 MHz, CDCl_3_) δ 8.43 (d, *J* = 8.7 Hz, 1H), 8.09 (s, 1H), 7.83 (s, 1H), 7.73 (d, *J* = 8.2 Hz, 2H), 7.63 (d, *J* = 8.6 Hz, 2H), 6.46 (d, *J* = 8.7 Hz, 1H), 4.87 (d, *J* = 5.8 Hz, 2H), 4.82 (s, 2H), 4.57–4.54 (m, 2H), 3.87–3.81 (m, 4H), 3.65–3.62 (m, 2H), 3.58 (s, 4H), 3.45 (s, 3H). ^13^C NMR (75 MHz, CDCl_3_) δ 172.38, 151.27, 144.51, 144.03, 143.81, 142.77, 136.66, 133.47, 124.33, 124.01, 122.85, 118.42, 112.72, 99.77, 71.49, 70.43, 70.31, 69.94, 69.24, 50.53, 39.57, 28.32. HRMS [M+Na]^+^: *m/z* 616.2003 (+1.6 ppm).

**LTRZ7-Fluo:**N-(4-cyanophenyl)-2-(2-(2-(2-(4-(((7-nitrobenzo[c][1,2,5]oxadiazol-4-yl)amino)methyl)-1H-1,2,3-triazol-1-yl)ethoxy)ethoxy)ethoxy)acetamide

Orange solid (yield = 69 %). ^1^H NMR (300 MHz, CDCl_3_) δ 8.95 (s, 1H), 8.45 (s, 1H), 7.74 (d, *J* = 8.0 Hz, 2H), 7.60 (d, *J* = 8.0 Hz, 2H), 6.39 (s, 1H), 4.82 (bs, 1H), 4.59 (s, 2H), 4.16 (s, 2H), 3.92 (s, 2H), 3.76 (s, 2H), 3.63 (s, 6H), 1.25 (s, 2H). HRMS [M+Na]^+^: *m/z* 574.1766 (-1.6 ppm).

**^1^H NMR data of final compounds**

**TRZ1:**

^1^H NMR (300 MHz, DMSO-D_6_): δ 7.43-7.17 (m, 17H), 6.73 (d, J= 8.5 Hz, 2H), 4.38 (s, 5H), 3.22 (s, 3H).

**TRZ2:**

^1^H NMR (300 MHz, DMSO-D_6_): δ 7.98-7.86 (m, 4H), 7.58-7.45 (m, 14H), 7.43-7.30 (m, 4H), 6.77 (d, J= 8.6 Hz, 2H), 4.95 (s, 5H), 2.88 (t, J= 7.2 Hz, 2H), 2.79-2.59 (m, 4H), 2.08 (s, 3H), 1.73-1.57 (m, 2H), 0.94 (t, J= 7.4 Hz, 3H).

**TRZ3:**

^1^H NMR (300 MHz, DMSO-D_6_): δ 7.95-7.86 (m, 4H), 7.57-7.46 (m, 12H), 7.39-7.29 (m, 4H), 6.28 (d, J= 8.8 Hz, 2H), 6.14 (d, J= 8.8 Hz, 2H), 4.87 (s, 5H), 2.79-2.53 (m, 4H).

**TRZ4:**

^1^H NMR (300 MHz, DMSO-D_6_): δ 7.95-7.83 (m, 4H), 7.70 -7.59 (m, 3H), 7.55-7.31 (m, 18H), 7.24 (d, J= 8.7 Hz, 2H), 6.72 (d, J= 8.6 Hz, 2H), 4.93 (s, 5H), 3.50 (s, 3H), 2.76-2.59 (m, 4H).

**TRZ5:**

^1^H NMR (300 MHz, DMSO-D_6_): δ 8.01-7.83 (m, 4H), 7.60-7.45 (m, 12H), 7.43-7.28 (m, 4H), 7.18 (d, J= 8.6 Hz, 2H), 6.53 (d, J= 8.7 Hz, 2H), 4.92 (s, 5H), 3.52 (s, 3H), 3.16 (s, 3H), 2.81-2.56 (m, 4H).

**TRZ6:**

^1^H NMR (300 MHz, DMSO-D_6_): δ 10.28 (s, 1H), 7.96-7.84 (m, 4H), 7.56-7.43 (m, 14H), 7.40-7.30 (m, 4H), 6.53 (d, J= 8.4 Hz, 2H), 4.90 (s, 5H), 3.70-3.62 (m, 2H), 3.58-3.39 (m, 12H), 2.77-2.51 (m, 8H).

**TRZ7:**

^1^H NMR (300 MHz, DMSO-D_6_): δ 10.20 (s, 1H), 7.96-7.85 (m, 4H), 7.56-7.46 (m, 14H), 7.41-7.31 (m, 4H), 6.51 (d, J= 8.8 Hz, 2H), 4.91 (s, 5H), 2.76-2.56 (m, 4H), 2.27 (t, J= 7.3 Hz, 2H), 1.56 (sext, J= 7.3 Hz, 2H), 0.88 (t, J= 7.4 Hz, 3H).

**TRZ8:**

^1^H NMR (300 MHz, DMSO-D_6_): δ 9.98 (s, 1H), 7.96-7.85 (m, 4H), 7.59-7.47 (m, 14H), 7.40-7.31 (m, 4H), 6.54 (d, *J*= 8.7 Hz, 2H), 4.91 (s, 5H), 4.08 (s, 2H), 3.69-3.49 (m, 10H), 3.41-3.33 (m, 2H), 2.76-2.57 (m, 4H).

**TRZ2-PEG:**

^1^H NMR (300 MHz, DMSO-D_6_): δ 7.95-7.90 (m, 3H), 7.54-7.48 (m, 16H), 7.39-7.35 (m, 3H), 6.75 (d, J = 8.6 Hz, 2H), 4.95 (s, 5H), 4.82-4.73 (m, 4H), 3.72-3.64 (m, 2H), 3.63-3.57 (m, 4H), 3.56 (s, 3H), 3.41-3.33 (m, 2H), 2.75- 2.56 (m, 6H).

**TRZ2-PEG-Fluo:**

^1^H NMR (300 MHz, DMSO-D_6_): δ 9.85 (s, 1H), 8.56-8.42 (m, 1H), 8.13-8.07 (m, 1H), 7.96-7.89 (m, 3H), 7.77-7.68 (m, 1H), 7.54-7.47 (m, 14H), 7.39-7.33 (m, 4H), 6.72 (d, J= 8.7 Hz, 2H), 6.51 (d, J= 9.0 Hz, 1H), 4.95 (s, 5H), 4.71-4.69 (m, 2H), 4.49 (t, J= 5.1 Hz, 2H), 3.79 (t, J= 5.1 Hz, 2H), 3.65-3.59 (m, 2H), 3.53-3.45 (m, 8H), 3.27 (s, 3H), 2.76-2.62 (m, 4H).

**TRZ7-Fluo:**

^1^H NMR (300 MHz, DMSO-D_6_): δ 9.96 (s, 1H), 9.84 (t, J= 6.0 Hz, 1H), 8.48 (d, J= 8.9 Hz, 1H), 8.09 (s, 1H), 7.93-7.86 (m, 4H), 7.83-7.70 (m, 1H), 7.54-7.48 (m, 14H), 7.39-7.32 (m, 4H), 6.51 (dd, J= 8.8, 1.4 Hz, 2H), 4.91 (s, 5H), 4.81-4.68 (m, 2H), 4.48 (t, J= 5.2 Hz, 2H), 4.08-4.01 (m, 2H), 3.78 (t, J= 5.2 Hz, 2H), 3.60-3.53 (m, 2H), 3.51-3.43 (m, 6H), 2.76-2.57 (m, 4H).

**Supplementary Table**

**Table S1.** Crystal data and structure refinement parameters for Ru-TRZ hybrids, TRZ2, TRZ5 and TRZ7 compounds.

| Compound | TRZ2 | TRZ5 | TRZ7 |
| --- | --- | --- | --- |
| Recrystallyzation | Acetone/n-hexane | DCM/n-hexane | DCM/n-hexane |
| Empirical formula | C_44_H_43_F_3_N_4_O_4_P_2_RuS**^.^**(CH_3_)_2_CO | C_41_H_39_F_3_N_4_O_3_P_2_RuS | C_43_H_40_F_3_N_2_O_4_P_2_RuS |
| *T*/K | 150(2) | 150(2) | 150(2) |
| Formula weight | 1001.97 | 887.83 | 900.84 |
| Crystal system | Monoclinic | Monoclinic | Monoclinic |
| Space Group | *P2_1_/n* | *P2_1_/n* | *Cc* |
| *a*/Å | 15.9780(15) | 14.6751(11) | 10.9954(10) |
| *b*/Å | 15.4451(16) | 14.4501(11) | 20.0013(17) |
| *c*/Å | 18.9527(19) | 18.7472(13) | 17.8567(12) |
| *β*/deg | 99.737(5) | 101.947(3) | 93.572(4) |
| *V*/Å^3^ | 4609.8(8) | 3889.4(5) | 3919.5(6) |
| *Z* | 4 | 4 | 4 |
| *ρ*_calc_/g⋅cm^-3^ | 1.444 | 1.516 | 1.527 |
| *µ*/mm^-1^ | 0.517 | 0.598 | 0.596 |
| *F*(000) | 2064 | 768 | 1844 |
| *θ* limits/deg | 2.686 to 27.251 | 2.426 to 27.530 | 2.036 to 26.142 |
| Limiting indices | –20 ≤ *h* ≤ 20 | –19 ≤ *h* ≤ 14 | –11 ≤ *h* ≤ 13 |
|  | –19 ≤ *k* ≤ 14 | –15 ≤ *k* ≤ 18 | –21 ≤ *k* ≤ 24 |
|  | –24 ≤ *l* ≤ 23 | –24 ≤ *l* ≤ 24 | –22 ≤ *l* ≤ 22 |
| No. of refns collected/unique | 40492 / 10234;  [R(int) = 0.0360] | 35738 / 8938;  [R(int) = 0.0415] | 15021 / 6687;  [R(int) = 0.0418] |
| Completeness to *θ* | 99.8 % | 99.9 % | 99.8 % |
| Data / restraints / parameters | 10234 / 0 / 569 | 8938 / 0 / 496 | 6687 / 2 / 505 |
| GOF on *F*^2^ | 1.061 | 1.071 | 0.899 |
| Final *R* indices [*l* > 2σ(*l*)] | *R1* = 0.0354, *wR*2 = 0.0978 | *R1* = 0.0274*, wR2 =* 0.0704 | *R1* = 0.0383*, wR2* = 0.0981 |
| *R* indices (all data) | R1 = 0.0473, wR2 = 0.1043 | *R1 = 0.0336, wR2 = 0.0728* | *R1* = 0.0436*, wR2* = 0.1019 |
| Largest diff peak and hole/*e*⋅Å^-3^ | 0.731 and -0.589 | 0.614 and -0.574 e.A^-3 | 1.081 and -0.874 |

**Supplementary Figures**

**Figure S1. ^1^H-NMR (300 MHz, DMSO-D_6_) spectrum of TRZ1.**

**Figure S2.** **^1^H-NMR (300 MHz, DMSO-D_6_) spectrum of TRZ2.**

**Figure S3.** **^1^H-NMR (300 MHz, DMSO-D_6_) spectrum of TRZ3.**

**Figure S4.** **^1^H-NMR (300 MHz, DMSO-D_6_) spectrum of TRZ4.**

**Figure S5.** **^1^H-NMR (300 MHz, DMSO-D_6_) spectrum of TRZ5.**

**Figure S6.** **^1^H-NMR (300 MHz, DMSO-D_6_) spectrum of TRZ6.**

**Figure S7.** **^1^H-NMR (300 MHz, DMSO-D_6_) spectrum of TRZ7.**

**Figure S8.** **^1^H-NMR (300 MHz, DMSO-D_6_) spectrum of TRZ8.**

**Figure S9.** **^1^H-NMR (300 MHz, DMSO-D_6_) spectrum of TRZ2-PEG.**

**Figure S10.** **^1^H-NMR (300 MHz, DMSO-D_6_) spectrum of TRZ2-PEG-Fluo.**

**Figure S11.** **^1^H-NMR (300 MHz, DMSO-D_6_) spectrum of TRZ7-Fluo.**

**Figure S12. Full and uncropped Western blots relative to Fig. 3.**

**Figure S13. Full and uncropped Western blots relative to Fig. 4.**

**Figure S14. Full and uncropped Western blots relative to Fig. 4.**

**Figure S15**. **Ru-TRZ exhibit cytotoxicity against HT29 cells in a dose-dependent manner.** HT29 cells were exposed for 24 h to vehicle-control DMSO or several concentrations of Ru-TRZ using a serial dilution strategy. Cell death was assessed by the AK release assay. Dose-response curves were determined for (A) TRZ2, (B) TRZ7, (C) TRZ2-PEG, and (D) TRZ2-PEG-Fluo. Results are from at least three independent experiments.

**Figure S16.** **Ru-TRZ hybrids induce necroptotic cell death in L929 cells**. L929 cells were treated with indicated Ru-TRZ (10 µM), alone or co-incubated with Nec-1 (necroptosis inhibitor) and the pan caspase inhibitor Z-VAD (apoptosis inhibitor). Mouse TNF-α was used as positive control and DMSO as vehicle control. Cell death and cell viability were assessed after 24 h of incubation with compounds using a luminescence-based readout for AK release, and a colorimetric-based readout for MTS metabolism. Results are expressed as mean ± SEM fold-change to control, from at least three independent experiments. ^#^*p* < 0.05 and ^§^*p* < 0.001 from control vehicle-treated cells; **p* < 0.05 and ^$^*p* < 0.001 from cells exposed to Ru-TRZ alone.
